# Sex differences in the associations of placental epigenetic aging with fetal growth

**DOI:** 10.18632/aging.102124

**Published:** 2019-08-08

**Authors:** Fasil Tekola-Ayele, Tsegaselassie Workalemahu, Gezahegn Gorfu, Deepika Shrestha, Benjamin Tycko, Ronald Wapner, Cuilin Zhang, Germaine M. Buck Louis

**Affiliations:** 1 Epidemiology Branch, Division of Intramural Population Health Research, National Institute of Child Health and Human Development, National Institutes of Health, Bethesda, MD 20892, USA; 2 Department of Clinical Laboratory Science, College of Nursing and Allied Health Sciences, Howard University, Washington, DC 20059, USA; 3 Hackensack-Meridian Health Center for Discovery and Innovation and the Hackensack-Meridian Health School of Medicine at Seton Hall University, Nutley, NJ 07110, USA; 4 Department of Obstetrics and Gynecology, Columbia University, New York, NY 10032, USA; 5 Dean’s Office, College of Health and Human Services, George Mason University, Fairfax, VA 22030, USA

**Keywords:** epigenetic clock, placental aging, fetal growth, sex differences, developmental origins of health and disease (DOHaD)

## Abstract

Identifying factors that influence fetal growth in a sex-specific manner can help unravel mechanisms that explain sex differences in adverse neonatal outcomes and *in-utero* origins of cardiovascular disease disparities. Premature aging of the placenta, a tissue that supports fetal growth and exhibits sex-specific epigenetic changes, is associated with pregnancy complications. Using DNA methylation-based age estimator, we investigated the sex-specific relationship of placental epigenetic aging with fetal growth across 13-40 weeks gestation, neonatal size, and risk of low birth weight. Placental epigenetic age acceleration (PAA), the difference between DNA methylation age and gestational age, was associated with reduced fetal weight among males but with increased fetal weight among females. PAA was inversely associated with fetal weight, abdominal circumference, and biparietal diameter at 32-40 weeks among males but was positively associated with all growth measures among females across 13-40 weeks. A 1-week increase in PAA was associated with 2-fold (95% CI 1.2, 3.2) increased odds for low birth weight and 1.5-fold (95% CI 1.1, 2.0) increased odds for small-for-gestational age among males. In all, fetal growth was significantly reduced in males but not females exposed to a rapidly aging placenta. Epigenetic aging of the placenta may underlie sex differences in neonatal outcomes.

## Introduction

Fetal growth is an important predictor of neonatal and infant morbidity and mortality [1], childhood morbidity [2,3], and is a risk factor for adult diseases including type 2 diabetes, obesity, cardiovascular diseases and cognitive dysfunction [4–6]. Accumulating evidence has shown that there are sex differences in fetal growth [7], the risk for adverse birth outcomes [8,9], and the incidence and severity of cardiometabolic diseases in later life [10]. Several studies have found that male fetuses are more vulnerable to adverse neonatal outcomes than female fetuses [11–14]. Identifying factors that influence fetal growth in a sex-specific manner can help unravel mechanisms that explain sex differences in adverse neonatal outcomes and the early origins of cardiovascular disease disparities by sex. In the long run, this knowledge is crucial for developing strategies that facilitate equitable health care to men and women.

The molecular mechanisms that may underlie sex differences in fetal growth during *in-utero* environmental alternations may best be studied in the placenta, a transient organ at the maternal-fetal interface that plays a key role in fetal growth and development [15]. Compromised placental function has often been associated with aberrant fetal growth and risk of low birth weight [16,15]. Male fetuses have more severe placental histopathological lesions [17] and higher placental production of endotoxin-induced tumor necrosis factor-response than female fetuses [18]. Moreover, placental response to adverse perinatal exposures [19,20] and placental epigenomic and transcriptomic profiles vary by fetal sex [19,21–23].

To support the demands of the growing fetus, the placenta undergoes physiologic developmental changes [24,25]; however, some placentas experience aging earlier than others [24,25]. Studies that used placental trophoblast telomere shortening and ultrasonographic calcification to determine placental tissue aging found that early placental aging increases the risk of several adverse fetal outcomes including fetal growth restriction and low birthweight [26–30]. Accelerated tissue aging may be induced by stressors including short or dysfunctional telomeres, oxidative stress, DNA damage, and epigenetic disruption [25,29,31–34]. Recently, DNA methylation profiles at selected cytosine-phosphate-guanine genomic sites (CpGs) have been used as epigenetic clocks to predict the biological age of tissues with high accuracy [35,36]. Epigenetic age acceleration, defined as the difference between DNA-methylation age and chronological age, in blood has successfully predicted aging-related conditions, adult mortality, life span [36,37], and birthweight [38–40]. In pregnancy, Mayne et al. predicted placental epigenetic age with high accuracy using DNA methylation at 62 CpGs and found that placental epigenetic age acceleration (PAA), i.e., the difference between placental DNA methylation age and gestational age at delivery, is associated with early onset preeclampsia [41]. Maternal dyslipidemia in early pregnancy has been associated with accelerated epigenetic aging of the placenta in a sex-dependent manner [42].

Emerging data suggest that the epigenetic aging rate may be largely determined in early pregnancy. For example, twin studies reported considerably high heritability (57%) of epigenetic age acceleration in the placenta [41] as well as other tissues [35,36]. In addition, the rate of epigenetic aging has been found to be developmentally adjusted [36,43] and remains stable across the lifespan [44]. In the present study, we investigated sex-specific associations of PAA with fetal growth, neonatal anthropometry measures, and risk of low birth weight in a cohort of pregnant women in the U.S. with high quality longitudinal fetal sonography data. Specifically, we tested associations of PAA with: 1) longitudinal trajectories of fetal growth measures (i.e., fetal weight, head circumference, biparietal diameter, abdominal circumference, humeral length, and femur length) across 13-40 weeks gestation; 2) fetal growth measures at each gestation week; 3) neonatal anthropometry (birth weight, birth length, head circumference, and abdominal circumference); and 4) risk of low birth weight (birthweight less than 2500 g) and small-for-gestational age (SGA: birthweight less than the 10^th^ percentile for gestation age) at birth.

## RESULTS

Male fetuses had significantly larger head circumference, biparietal diameter, and femur length at three gestational age time points: 13 weeks and 6 days, 27 weeks and 6 days, and 40 weeks and 0 days in comparison to female fetuses (*p* <0.05). Male neonates had significantly longer birth lengths (*p* = 0.01) and larger head circumferences (*p* = 0.002) than female neonates. A higher percentage of male than female neonates had low birth weights (9.9% *vs*. 4.7%), although the difference was not significant (*p* = 0.08). There were no significant sex differences in gestational age, DNA methylation age, or PAA (Table 1). There were no significant sex differences in placental weight or presence of calcification (Supplementary Table 1).

**Table 1:** Fetal and neonatal characteristics of study participants.

	**Male fetus (n=152)**	**Female fetus (n=149)**	***p***
**Mean ± s.d.**			
Gestational age, weeks	39.4 ± 0.1	39.6 ± 0.1	0.15
DNA methylation age, weeks	35.6 ± 1.4	35.7 ± 1.6	0.63
Placental age acceleration, weeks	-3.8 ± 1.6	-3.9 ± 1.8	0.59
Fetal weight, g			
13 weeks gestation	81.6±8.1	81.4±7.9	0.86
27 weeks gestation	1060.5±129.0	1064.2±118.6	0.52
40 weeks gestation	3235.4±485.6	3271.0±466.0	0.52
Head circumference, mm			
13 weeks gestation	94.7±6.1	93.8±5.7	0.16
27 weeks gestation	256.1±7.8	253.7±7.6	0.01
40 weeks gestation	330.9±10.5	327.4±10.9	0.004
Biparietal diameter, mm			
13 weeks gestation	25.6±1.7	25.3±1.6	0.12
27 weeks gestation	69.1±2.5	68.6±2.4	0.04
40 weeks gestation	91.6±3.0	91.1±2.9	0.14
Abdominal circumference, mm			
13 weeks gestation	75.2±5.3	74.4±4.9	0.21
27 weeks gestation	229.0±11.2	228.6±10.8	0.79
40 weeks gestation	342.8±19.2	344.1±19.8	0.56
Humeral length, mm			
13 weeks gestation	12.2±1.8	12.2±1.7	0.95
27 weeks gestation	46.4±1.8	46.4±1.6	0.95
40 weeks gestation	63.2±1.9	63.2±1.7	0.83
Femur length, mm			
13 weeks gestation	11.6±1.7	11.8±1.8	0.27
27 weeks gestation	50.7±1.5	51.1±1.5	0.02
40 weeks gestation	72.5±1.3	72.9±1.3	0.01
Birth weight, g	3196.7±489.6	3133.0±405.8	0.22
Birth length, cm	49.8±2.4	49.1±2.2	0.01
Head circumference, cm	33.9±1.4	33.4±1.4	0.002
Abdominal circumference, cm	32.6±2.1	32.5±2.1	0.69
**n (%)**			
Low birth weight	15 (9.9)	7 (4.7)	0.08
Small-for-gestational age	28 (18.4)	29 (19.5)	0.82

### Sex-specific associations between PAA and longitudinal fetal growth

**Figure 1:**
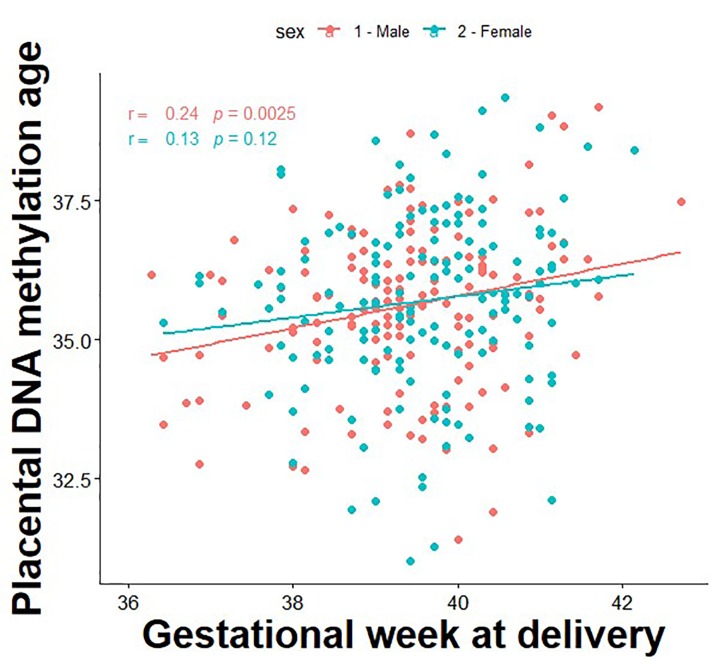
**Correlation between placental DNA methylation age and gestational age at birth.**

The correlation between DNA methylation age and gestational age is presented in Figure 1. First, we tested associations between a 1-week increase in PAA and longitudinal trajectories of fetal growth by sex. PAA was found to be significantly associated with decreased fetal weight among males (mean difference [95% CI]: -17.4 g [-34.0, -0.82]), whereas positive associations were found in females for fetal weight (14.5 g [0.9, 28.1]), head circumference (1.2 mm [0.5, 1.8]), biparietal diameter (0.4 mm [0.2, 0.6]), abdominal circumference (1.3 mm [0.4, 2.3]), humeral length (0.2 mm [0.1, 0.4]), and femur length (0.2 mm [0.1, 0.3]) (Table 2).

**Table 2:** Sex-specific change in longitudinal fetal growth measures per one week increase in placental age acceleration.

**Fetal growth measure**	**Male fetus**			**Female fetus**	
	**Mean difference (95% CI)**	***p***		**Mean difference (95% CI)**	***p***
Fetal weight, g	-17.4 (-34.0, -0.8)	0.04		14.5 (0.9, 28.1)	0.04
Head circumference, mm	-0.2 (-0.9, 0.6)	0.68		1.2 (0.5, 1.8)	0.001
Biparietal diameter, mm	-0.2 (-0.4, 0.1)	0.21		0.4 (0.2, 0.6)	8.5e-5
Abdominal circumference, mm	-0.8 (-1.9, 0.3)	0.16		1.3 (0.4, 2.3)	0.01
Humeral length, mm	-0.0 (-0.2, 0.2)	0.85		0.2 (0.1, 0.4)	0.01
Femur length, mm	0.0 (-0.2, 0.2)	0.97		0.2 (0.1, 0.3)	0.004

Adjusted for maternal age, pre-pregnancy body mass index, race/ethnicity, marital status, educational status, health insurance ownership, parity, and mode of onset of labor.

### Sex-specific associations between PAA and fetal size at each gestational week

**Figure 2:**
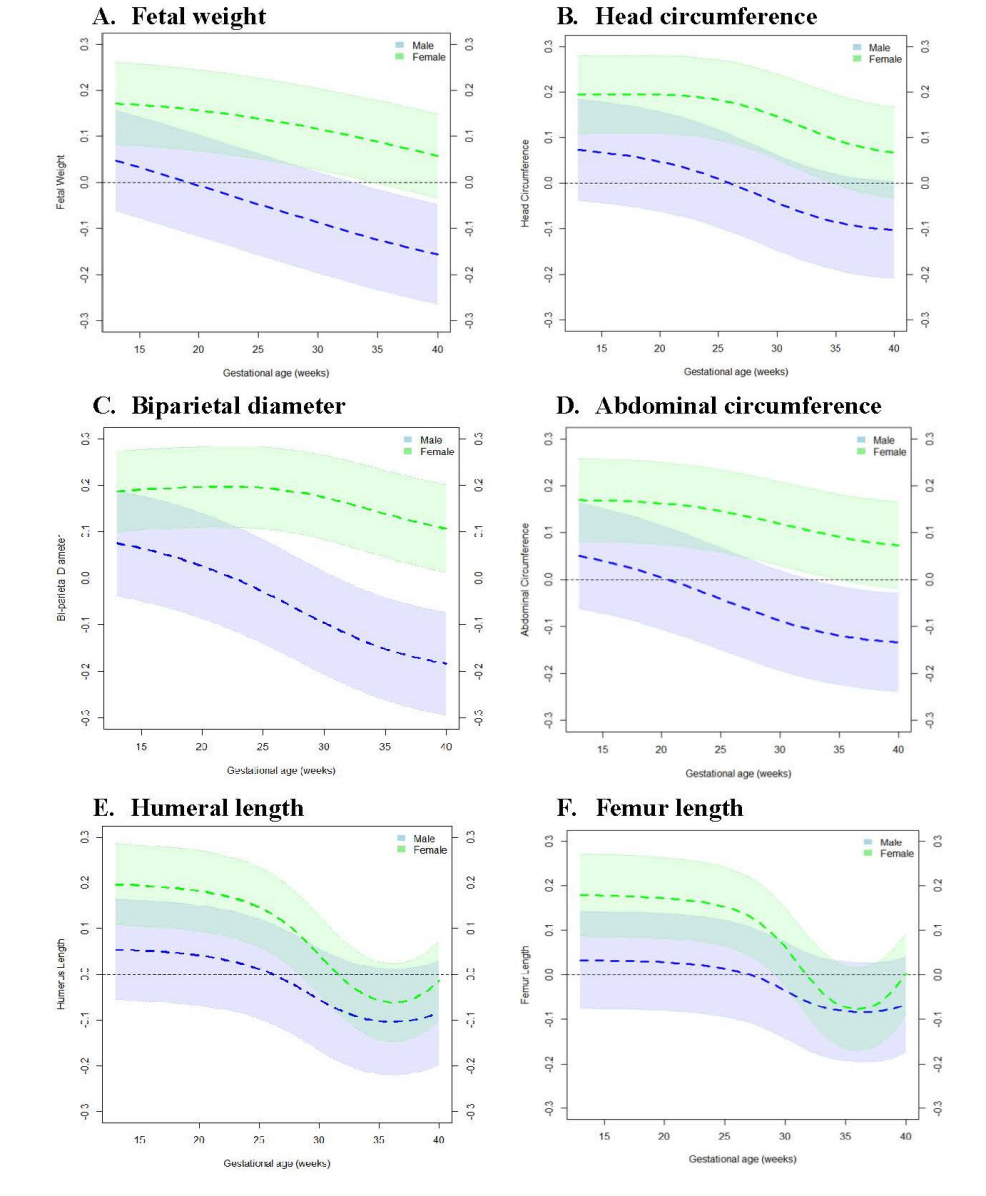
**Change in fetal growth measure *z*-score at 13-40 weeks’ gestation per one week increase in placental age acceleration.** Models were adjusted for maternal age, pre-pregnancy body mass index, race/ethnicity, marital status, educational status, health insurance ownership, parity, and mode of onset of labor. Black horizontal line along the 0-mark on the vertical axis represents the null. Dotted lines in color shaded areas represent the lower and upper bounds of 95% Confidence Intervals. (**A**) Fetal weight. (**B**) Head circumference. (**C**) Biparietal diameter. (**D**) Abdominal circumference. (**E**) Humeral length. (**F**) Femur length.

To determine the specific window of gestation in which associations between PAA and fetal growth measures exhibit sex-specific differences, we tested the relationship between PAA and fetal growth at each week of gestation across 13-40 gestational weeks. Figure 1 shows that among males, PAA and fetal growth were inversely associated with even stronger relationships observed at advancing gestational ages. However, among females, PAA and fetal growth were positively associated in early gestation, but the relationships became weaker with advancing gestational ages (Figure 2). More specifically, among males, PAA was significantly inversely associated with fetal weight at 34-40 weeks, biparietal diameter at 32-40 weeks, and abdominal circumference at 33-40 weeks gestation. In contrast, among females, PAA was significantly positively associated with fetal weight at 13-34 weeks, head circumference at 13-34 weeks, biparietal diameter at 13-40 weeks, abdominal circumference at 13-34 weeks, humeral length at 13-28 weeks, and femur length at 13-29 weeks gestation (Supplementary Table 2-7).

### Sex-specific associations of PAA with neonatal size and risk of low birth weight

**Figure 3:**
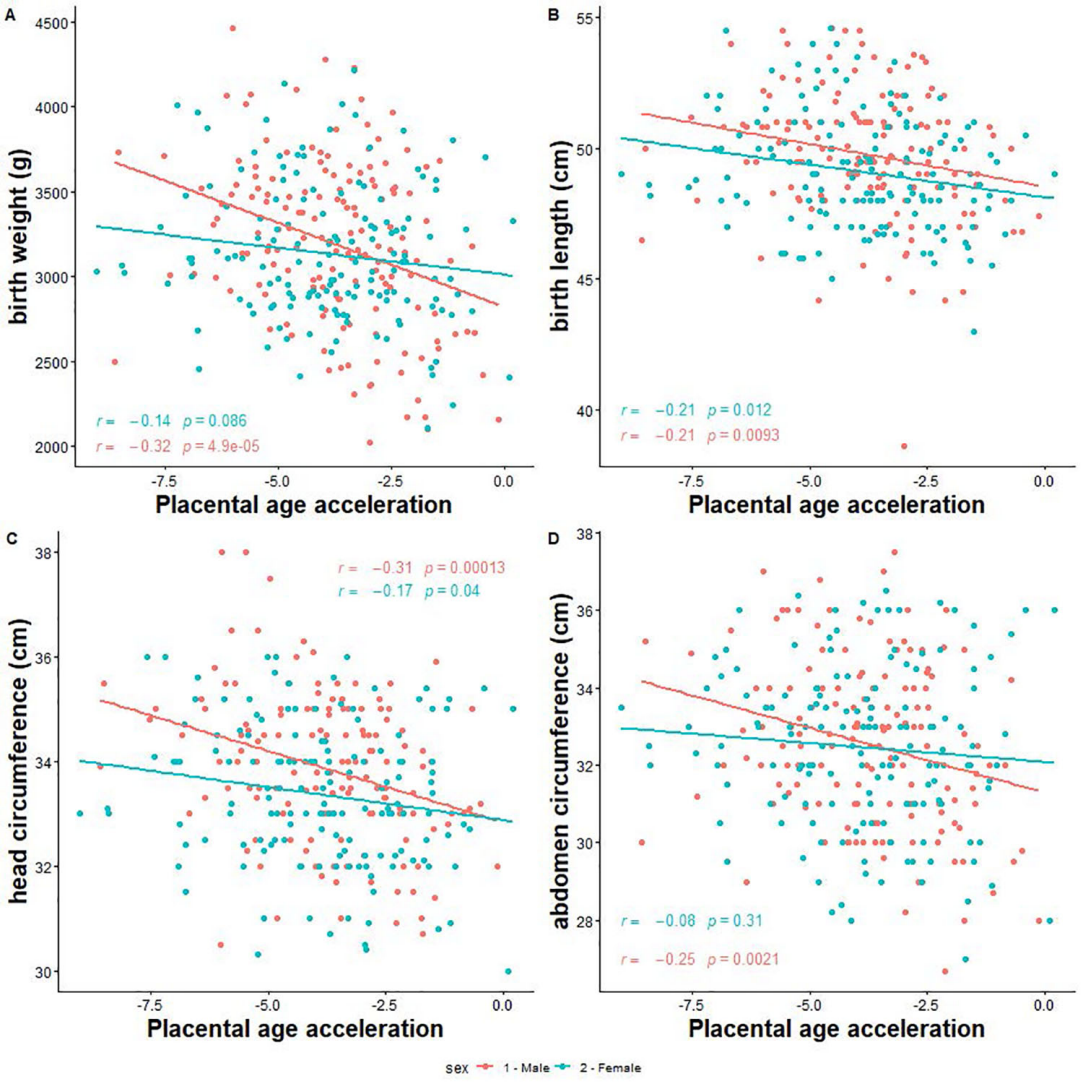
**Correlation between placental epigenetic age acceleration and neonatal anthropometry measures. (A**) Birth weight. (**B**) Birth length. (**C**) Head circumference. (**D**) Abdominal circumference.

Next, we investigated the associations of PAA with neonatal size, and risk of low birth weight and SGA. We found that a 1-week increase in PAA was significantly associated with 114 g ([95% CI -166.1, -61.9] smaller birth weight, 0.4 cm [-0.7, -0.1] shorter birth length, 0.3 cm [-0.5, -0.2] smaller head circumference, 0.4 cm [-0.6, -0.2] smaller abdominal circumference among male neonates (Table 3, Figure 3). Moreover, a 1-week increase in PAA was associated with 2.0 times [1.2, 3.2] increased odds for low birth weight and 1.5 times [1.1, 2.0] increased odds for SGA among males only. The corresponding associations among females were significant for birth length only (mean difference = -0.3 cm [-0.5, -0.1]) (Table 3).

**Table 3:** Sex-specific change in neonatal anthropometry measures per one week increase in placental age acceleration.

	**Male neonate (n=152)**			**Female neonate (n=149)**	
**Neonatal size**	**Estimate (95% CI)**	***p***		**Estimate (95% CI)**	***p***
Birth weight, g	-114.0 (-166.1, -61.9)	3.0e-5		-31.9 (-70.2, 6.4)	0.10
Birth length, cm	-0.4 (-0.7, -0.1)	0.004		-0.3 (-0.5, -0.1)	0.01
Head circumference, cm	-0.3 (-0.5, -0.2)	2.7e-5		-0.1 (-0.2, 0.0)	0.10
Abdominal circumference, cm	-0.4 (-0.6, -0.2)	0.001		-0.1 (-0.3, 0.1)	0.39
Low birth weight	Reference	0.01		Reference	0.11
	2.0 (1.2, 3.2)			1.7 (0.9, 3.0)	
Small-for-gestational age	Reference	0.02		Reference	0.28
	1.5 (1.1, 2.0)			1.2 (0.9, 1.5)	

Adjusted for maternal age, pre-pregnancy body mass index, race/ethnicity, marital status, educational status, health insurance ownership, parity, and mode of onset of labor. Estimate refers to mean difference except for low birthweight and small-or-gestational-age in which it stands for odds ratio (OR).

Statistically significant effect modification by fetal sex was observed for birth weight (*p-*for-interaction = 0.01), head circumference (*p*-for-interaction = 0.03), and abdominal circumference (*p*-for-interaction = 0.04).

## DISCUSSION

To our knowledge, this is the first study to investigate the relationships of placental epigenetic age acceleration with fetal growth, neonatal anthropometry measures, and risk of low birth weight. Overall, we found significant sex differences in the relationships between PAA and fetal growth. Among males, higher PAA was significantly associated with smaller fetal weight, biparietal diameter, and abdominal circumference from mid-third trimester through end of third trimester of pregnancy. A one-week increase in PAA was significantly associated with nearly two-fold increased risk of low birth weight and smaller neonatal anthropometry measures among males. However, among females, higher PAA was significantly associated with larger head bones (i.e., head circumference and biparietal diameter) from first trimester through end of third trimester, fetal weight and abdominal circumference from first trimester through mid-third trimester, and long bones (i.e., humeral and femur lengths) from first trimester through early third trimester.

The fetal sex-dependent relationship between fetal growth and PAA is likely to result from a combination of different mechanisms that operate in concert. Premature aging of the placenta may lead to placental insufficiency [30], which is associated with an increased risk of low birth weight [15,16,45,46 ] that is more common among male than female births [47,48]. Therefore, placental dysfunction resulting from accelerated epigenetic aging processes may be more severe in male than female fetuses, explaining the male sex-biased effect of accelerated placental aging on reduced fetal growth. We also observed that the effect of PAA on reduced fetal growth was stronger later in gestation during a time period associated with greater fetal demand for oxygen and nutrients [49]. Male fetuses have higher *in-utero* energy demand than female fetuses [50] and, thereby, may be more sensitive to PAA than female fetuses especially in context of a compromised placenta.

Previous studies suggest that female fetuses adopt risk-averse strategies by investing more resources in extra-embryonic tissue development that promotes survival in the event of environmental adversity [51,52]. For example, higher placental global DNA methylation and tighter regulation of gene expression have been reported in female than male fetuses [52]. Moreover, genes that encode the luteinizing and human chorionic gonadotropin hormones that promote placental growth have higher expression in female than male placentas [51]. As such, our observation that female fetal growth was enhanced in early gestation with fast-aging placenta is consistent with these studies. Our findings also indicate the possibility that females employ a more effective adaptive responses to enhance survival against the ensuing accelerated aging that leads to decreased growth in later gestation.

The positive association between PAA and long bone lengths among female fetuses was significant until early third trimester in contrast to head and abdominal circumference that remained significant through mid- to late- third trimester. Moreover, birth length was inversely associated with PAA among females. Previous studies have indicated that with pregnancies in which poor placental function may lead to fetal growth restriction, the fetus adopts a compensatory process that tends to favor growth of vital organs such as the brain, heart, and adrenals at the expense of other structures such as long bones, a process known as ‘brain sparing’ [53,54]. Our finding coincides with this and further suggests that females may have a more successful adaptive strategy that prioritizes growth of the brain at the expense of skeletal growth in the presence of a rapidly aging placenta with compromised function.

We also found that with increasing gestational age, the positive relationship between PAA and fetal growth among females became weaker and the inverse relationship between PAA and fetal growth among males became stronger. Studies in mice and human placenta have found that the gene expression profile and maternal-fetal interface of the placenta undergo dramatic transitions at mid-gestation [55,56] to meet the demands of the rapidly growing fetus in later gestation. In addition, chronic oxidative stress builds-up with advancing gestational age [57,58]. The resulting progressive placental degeneration [57,58] and accumulation of environmentally-induced DNA methylation changes in the placenta [59,60] may lead to more compromised fetal growth in the second and third trimesters [57,58].

There are limitations to our study. All placenta samples were obtained at delivery during the third trimester (mean ± SD gestational age at delivery = 39.5±1.1 weeks), limiting the scope the study’s ability to make inference on the temporal relationship between PAA at different windows of gestation and fetal growth. Interestingly, however, emerging data suggest that the epigenetic aging “clock” of the placenta may be set largely in early pregnancy and remains largely stable across gestation, lending support to our findings of consistent relationships of PAA with neonatal as well as pre-natal anthropometry measures. For example, PAA has been found to be highly heritable (57%) [41] and the rate of epigenetic aging is found to be developmentally adjusted [36,43] and remains stable across the lifespan [44]. Together, these data suggest that PAA may not exhibit substantial changes across gestation. Future longitudinal studies are needed to evaluate the stability of PAA during gestation using biomarkers of placental aging in maternal blood obtained at serial gestation times.

Strengths of our study include the race/ethnic diversity of the study participants and high-quality longitudinal measurement of fetal biometry using a standardized ultrasonology protocol with established quality control after intensive training and credentialing of sonographers [61]. During recruitment, several major chronic diseases were used as exclusion criteria, minimizing confounding effect of unaccounted pre-pregnancy diseases that may influence fetal growth [62]. In addition, the low risk antenatal profiles of pregnant women in our cohort enhanced our ability to follow women through expected gestation with minimal loss of women to iatrogenic induction of labor for clinical indication. Continued improvements in tissue-specific epigenetic clock algorithms [63] will be valuable to refine the findings.

In summary, this study found associations between epigenetic aging of term placenta and fetal growth throughout pregnancy. Notably, placental epigenetic age acceleration was found to be associated with fetal growth in a sex-dependent manner, showing stronger fetal and neonatal size-lowering effect among male than female fetuses. This finding recommends future studies to investigate placental function in relation to fetal outcomes stratified by fetal sex. Future studies are needed to test the potential for using non-invasive maternal samples during pregnancy for predicting placental epigenetic aging, so that the prognostic and diagnostic use of the placental clock may be realized in clinical practice. Together with ongoing efforts that aim to uncover the mechanisms of epigenetic aging [64], this study helps advance our understanding of the biological underpinnings of sex differences in aberrant fetal growth and the early programming of adult diseases.

## MATERIALS AND METHODS

### Ethics statement

The study was approved by institutional review boards at NICHD and each of the participating clinical sites. Written informed consent was obtained from each woman who participated in the study.

### Data set

The study included 312 women who provided placenta samples at delivery as part of the *Eunice Kennedy Shriver* National Institute of Child Health and Human Development (NICHD) Fetal Growth Studies - Singletons. Women without major pre-existing medical conditions from each of four self-identified race/ethnic groups (i.e., non-Hispanic White, non-Hispanic Black, Hispanic, and Asian or Pacific Islander) were recruited from 12 clinic sites in the United States and were followed through delivery [62,65]. Details about the study design and data collection methods has been described previously [62,65]. Gestational age was determined using the date of the last menstrual period and confirmed by ultrasound between 8 weeks to 13 weeks and 6 days of gestation [65]. The study was approved by institutional review boards at NICHD and each of the participating clinical sites.

### Fetal growth and neonatal anthropometry measurements

The study implemented a standardized ultrasonology protocol with established quality control after intensive training and credentialing of sonographers [61]. After the first ultrasound to confirm gestational age, pregnant women underwent five standardized ultrasounds at *a priori* defined gestational ages. At each ultrasound visit, head circumference, biparietal diameter, abdominal circumference, femur length, and humerus length were measured. Estimated fetal weight was calculated from head circumference, abdominal circumference, and femur length using the Hadlock formula [66]. Neonates underwent standardized anthropometric measurements as previously described [67]. Low birth weight was defined as birth weight of less than 2500 g [68]. The study was approved by the institutional review boards of NICHD and each of the participating clinic sites. Written informed consent was obtained from all study participants.

### Placental DNA methylation measurement and DNA methylation age calculation

Placental samples were obtained within one hour of delivery. Placental parenchymal biopsies measuring 0.5 cm x 0.5 cm x 0.5 cm were taken from the fetal side, collected directly below the fetal surface of the placenta. Samples were placed in RNALater and frozen for molecular analysis. Processing of the placental biopsies was performed at the Columbia University Irving Medical Center and details have been described previously [69]. DNA from placental biopsies was extracted and assayed using Illumina’s Infinium Human Methylation450 Beadchip (Illumina Inc., San Diego, CA. Standard Illumina protocols were followed for background correction, normalization to internal control probes, and quantile normalization. The resulting intensity files were processed with Illumina’s GenomeStudio, which generated average beta-scores (i.e., the fraction of methylated sites per sample by taking the ratio of methylated and unmethylated fluorescent signals at each queried CpG) and detection *p* values (that characterize the chance that the target sequence signal was distinguishable from the negative controls).

Beta scores with an associated detection *p* values ≥0.05 were set to missing. In addition, probes with mean detection *p* value ≥0.05 (n=36), cross-reactive (n=24,491), non-autosomal (n=14,589), and CpG sites located within 20 base pair from known single nucleotide polymorphisms (SNPs) (n=37,360) were removed. After these QC procedures, methylation data were available for 409,101 CpGs. Placental DNA samples were also genotyped on HumanOmni2.5 Beadchips (Illumina Inc., San Diego, CA). A total of 11 samples showing discrepancies between phenotypic sex and genotypic sex (n=4), that were outliers from the distribution of the samples’ genetic clusters based on multi-dimensional scaling plots (n=6), and with a mismatching sample identifier (n=1) were excluded. Normalization was performed using the modified Beta MIxture Quantile dilation (BMIQ) method to correct the probe design bias in the Illumina Infinium Human Methylation450 Beadchip and achieve between-sample normalization [35,70]. Missing CpGs were imputed by the *k*-nearest neighbors method setting *k*=10. The tissue prediction of our samples was evaluated using Horvath’s “epigenetic clock” method [35]. All samples had high probability of prediction for placental tissue compared with all other tissues in the database.

Placental DNA methylation age was predicted using 62 CpGs that have previously been found to predict placental DNA methylation age with high accuracy [41]. DNA methylation age of each sample was determined by regressing the mean beta-values of the 62 CpGs by gestational age using an elastic net regression model [35]. Placental age acceleration (PAA) was defined to be the difference between placental DNA methylation age and gestational age at birth [41,35].

### Statistical analyses

Associations between PAA and longitudinal trajectories of fetal growth measures (i.e., fetal weight, head circumference, biparietal diameter, abdominal circumference, humeral length, and femur length) were tested using multiple linear mixed models with a random effect variable that corresponds to the mother-child pair to account for repeated measurements. The longitudinal fetal growth measures were included in the models after z-score transformation. Linear regression analyses were performed to test for associations between PAA and fetal growth measures at 13 to 40 weeks’ gestation and neonatal anthropometry (i.e., birth weight, birth length, head circumference, and abdominal circumference). Logistic regression analyses were performed to test for associations between PAA with risk of low birth weight and risk of SGA (birthweight less than the 10^th^ percentile for gestation age based on sex-specific birthweight references [71]). Effect modification of associations by neonatal sex was examined using stratified analyses and interaction terms in the regression models. All models were adjusted for maternal education (≤ high school vs. > high school), age, pre-pregnancy body mass index (BMI), race/ethnic group, marital status, health insurance, parity (nulliparous vs. ≥ 1 child), preeclampsia status, and mode of onset of labor (no labor vs spontaneous labor vs. induced labor). Unless specified otherwise, analyses were implemented using the software package R version 3.1.2 (R Development Core Team).

### Availability of Data

The data analyzed during the current study are available from the corresponding author on reasonable request. The placental DNA methylation data are available through dbGaP with accession number phs001717.v1.p1.

## SUPPLEMENTARY MATERIAL

Supplementary Tables
