# Supplementary material for: Sex differences in the associations of placental epigenetic aging with fetal growth
Source: Aging (Albany NY). 2019 Aug 8;11(15):5412–32. doi: 10.18632/aging.102124 (PMC6710059; doi:10.18632/aging.102124)
Supplement: Supplementary Tables [file aging-11-102124-s001.pdf]

SUPPLEMENTARY MATERIAL

Supplementary Tables

Supplementary Table 1. Additional characteristics of study participants.

Characteristics	Male	Female	*p*
Clinical site, n (%)			
Columbia University	26 (17.1%)	27 (18.1%)	0.70 [1]
Christina Care Health Systems	25 (16.4%)	27 (18.1%)	
Saint Peters University Hospital	26 (17.1%)	29 (19.5%)	
Medical University of South Carolina	14 (9.2%)	17 (11.4%)	
Northwestern University	11 (7.2%)	10 (6.7%)	
University of California Irvine	3 (2.0%)	2 (1.3%)	
Long Beach Memorial Medical Center	24 (15.8%)	17 (11.4%)	
New York Hospital Queens	8 (5.3%)	8 (5.4%)	
University of Alabama at Birmingham	6 (3.9%)	10 (6.7%)	
Fountain Valley Hospital	6 (3.9%)	1 (0.7%)	
Women and Infants Hospital of Rhode Island	3 (2.0%)	1 (0.7%)	
Parity, n (%)			
0	72 (47.4%)	68 (45.6%)	0.92 [1]
1	56 (36.8%)	52 (34.9%)	
2	18 (11.8%)	23 (15.4%)	
3	5 (3.3%)	5 (3.4%)	
>=4	1 (0.7%)	1 (0.7%)	
Maternal education, n (%)			
Less than high school	16 (10.5%)	21 (14.1%)	0.48 [2]
High school diploma or GED or equivalent	29 (19.1%)	27 (18.1%)	
Some college or Associate degree	42 (27.6%)	44 (29.5%)	
Bachelors' degree	41 (27.0%)	33 (22.1%)	
Masters' degree or Advanced degree	24 (15.8%)	24 (16.1%)	
Maternal age in years, median (Q1-Q3)	28.00 (23.50-31.50)	28.00 (23.00-32.00)	1.00 [2]
Gestational age at delivery in weeks, median (Q1-Q3)	39.43 (38.71-40.14)	39.71 (39.00-40.29)	0.16 [2]
Trimmed placental weight in g, median (Q1-Q3)	425.00 (355.00-488.00)	416.00 (365.00-489.00)	0.54 [2]
Placental disc length in cm, median (Q1-Q3)	20.00 (18.00-21.50)	20.50 (19.00-22.00)	0.05 [2]
Placental disc width in cm, median (Q1-Q3	17.00 (15.25-19.00)	17.00 (15.00-18.00)	0.32 [2]
Placental disc thickness in cm, median (Q1-Q3)	2.00 (1.40-2.50)	2.00 (1.30-2.35)	0.45 [2]
Placental maternal surface fibrin deposit, n (%)			
Absent	104 (69.3%)	111 (76.6%)	0.16 [2]
Present	46 (30.7%)	34 (23.4%)	
Placental maternal surface calcification, n (%)			
Absent	108 (71.1%)	87 (58.8%)	0.05 [2]
Present (Focal)	15 (9.9%)	27 (18.2%)	
Present (Diffuse)	29 (19.1%)	34 (23.0%)	

[1] based on Fisher's exact test; [2] based on Mann-Whitney test; Q1, 1st quartile value; Q3, 3rd quartile value

Supplementary Table 2. Change in fetal weight *z*-score at 13-40 weeks gestation per one week increase in placental age acceleration in male and female fetuses.

	Male				Female			
		95% CI				95% CI		
Gestational week	Beta	Lower	Upper	*p*	Beta	Lower	Upper	*p*
**13**	0.047	-0.062	0.157	0.40	0.172	0.082	0.261	**0.000261**
**14**	0.040	-0.069	0.149	0.48	0.170	0.081	0.259	**0.000281**
**15**	0.032	-0.077	0.142	0.56	0.168	0.080	0.257	**0.000308**
**16**	0.025	-0.085	0.134	0.66	0.166	0.078	0.255	**0.000345**
**17**	0.017	-0.093	0.126	0.76	0.164	0.076	0.253	**0.000394**
**18**	0.009	-0.100	0.119	0.87	0.162	0.074	0.250	**0.00046**
**19**	0.001	-0.108	0.111	0.98	0.159	0.071	0.247	**0.000547**
**20**	-0.007	-0.116	0.103	0.90	0.156	0.069	0.244	**0.000664**
**21**	-0.015	-0.124	0.095	0.79	0.153	0.066	0.241	**0.000822**
**22**	-0.023	-0.133	0.087	0.68	0.150	0.063	0.238	**0.001037**
**23**	-0.031	-0.141	0.079	0.58	0.147	0.059	0.234	**0.001332**
**24**	-0.039	-0.149	0.071	0.49	0.143	0.055	0.230	**0.001738**
**25**	-0.047	-0.157	0.062	0.40	0.139	0.051	0.226	**0.002302**
**26**	-0.055	-0.165	0.054	0.33	0.135	0.047	0.222	**0.003089**
**27**	-0.063	-0.173	0.046	0.26	0.130	0.043	0.218	**0.004193**
**28**	-0.071	-0.181	0.038	0.20	0.126	0.038	0.213	**0.005744**
**29**	-0.079	-0.188	0.030	0.16	0.121	0.033	0.209	**0.007925**
**30**	-0.087	-0.196	0.022	0.12	0.116	0.028	0.204	**0.010985**
**31**	-0.095	-0.204	0.015	0.09	0.111	0.023	0.199	**0.01526**
**32**	-0.102	-0.211	0.007	0.07	0.105	0.017	0.194	**0.021195**
**33**	-0.110	-0.219	-0.001	0.05	0.100	0.011	0.189	**0.029361**
**34**	-0.117	-0.226	-0.008	**0.037084**	0.094	0.005	0.183	**0.040468**
**35**	-0.124	-0.233	-0.015	**0.026975**	0.088	-0.001	0.178	0.06
**36**	-0.131	-0.240	-0.023	**0.019527**	0.082	-0.008	0.172	0.08
**37**	-0.138	-0.246	-0.029	**0.014098**	0.076	-0.014	0.167	0.10
**38**	-0.144	-0.252	-0.036	**0.010178**	0.070	-0.021	0.161	0.13
**39**	-0.150	-0.258	-0.042	**0.007366**	0.064	-0.028	0.155	0.17
**40**	-0.156	-0.264	-0.048	**0.005359**	0.057	-0.034	0.149	0.22

Adjusted for maternal age, pre-pregnancy body mass index, race/ethnicity, marital status, educational status, health insurance ownership, parity, and mode of onset of labor. Statistically significant associations ($p<0.05$) are marked in bold.

Supplementary Table 3. Change in head circumference *z*-score at 13-40 weeks gestation per one week increase in placental age acceleration in male and female fetuses.

	Male				Female			
		95% CI				95% CI		
Gestational week	Beta	Lower	Upper	*p*	Beta	Lower	Upper	*p*
**13**	0.073	-0.038	0.184	0.20	0.194	0.109	0.280	**1.87E-05**
**14**	0.070	-0.041	0.181	0.22	0.195	0.109	0.280	**1.76E-05**
**15**	0.068	-0.043	0.179	0.23	0.195	0.110	0.280	**1.67E-05**
**16**	0.065	-0.046	0.175	0.25	0.195	0.110	0.280	**1.6E-05**
**17**	0.061	-0.049	0.172	0.28	0.195	0.110	0.280	**1.56E-05**
**18**	0.057	-0.053	0.167	0.31	0.195	0.110	0.280	**1.56E-05**
**19**	0.053	-0.057	0.163	0.35	0.195	0.110	0.280	**1.6E-05**
**20**	0.047	-0.062	0.157	0.40	0.195	0.109	0.280	**1.72E-05**
**21**	0.042	-0.068	0.151	0.46	0.194	0.108	0.279	**1.95E-05**
**22**	0.035	-0.074	0.144	0.53	0.192	0.106	0.278	**2.38E-05**
**23**	0.028	-0.081	0.136	0.62	0.190	0.104	0.276	**3.14E-05**
**24**	0.019	-0.088	0.127	0.72	0.187	0.100	0.274	**4.58E-05**
**25**	0.010	-0.097	0.117	0.85	0.183	0.096	0.271	**7.4E-05**
**26**	0.000	-0.106	0.107	0.99	0.178	0.090	0.267	**0.000133**
**27**	-0.010	-0.116	0.096	0.85	0.172	0.082	0.261	**0.000262**
**28**	-0.021	-0.126	0.084	0.70	0.164	0.073	0.255	**0.000553**
**29**	-0.032	-0.137	0.073	0.55	0.156	0.063	0.248	**0.001219**
**30**	-0.043	-0.147	0.062	0.43	0.146	0.053	0.239	**0.002694**
**31**	-0.053	-0.157	0.051	0.32	0.136	0.041	0.230	**0.005771**
**32**	-0.063	-0.167	0.042	0.24	0.125	0.029	0.221	**0.011652**
**33**	-0.071	-0.176	0.033	0.18	0.115	0.018	0.211	**0.021754**
**34**	-0.079	-0.183	0.026	0.14	0.105	0.007	0.202	**0.037221**
**35**	-0.085	-0.190	0.020	0.11	0.096	-0.002	0.194	0.06
**36**	-0.091	-0.196	0.015	0.09	0.088	-0.011	0.186	0.08
**37**	-0.095	-0.200	0.011	0.08	0.081	-0.018	0.180	0.11
**38**	-0.098	-0.204	0.007	0.07	0.075	-0.024	0.175	0.14
**39**	-0.101	-0.206	0.005	0.07	0.071	-0.029	0.171	0.16
**40**	-0.102	-0.208	0.004	0.06	0.068	-0.032	0.168	0.18

Adjusted for maternal age, pre-pregnancy body mass index, race/ethnicity, marital status, educational status, health insurance ownership, parity, and mode of onset of labor. Statistically significant associations ($p<0.05$) are marked in bold.

Supplementary Table 4. Change in biparietal diameter *z*-score at 13-40 weeks gestation per one week increase in placental age acceleration in male and female fetuses.

	Male				Female			
		95% CI				95% CI		
Gestational week	Beta	Lower	Upper	*p*	Beta	Lower	Upper	*p*
**13**	0.075	-0.038	0.188	0.20	0.187	0.101	0.274	**4.26E-05**
**14**	0.070	-0.043	0.183	0.22	0.189	0.102	0.275	**3.67E-05**
**15**	0.064	-0.049	0.178	0.27	0.190	0.104	0.277	**3.18E-05**
**16**	0.058	-0.055	0.171	0.32	0.192	0.105	0.278	**2.76E-05**
**17**	0.051	-0.062	0.164	0.38	0.193	0.107	0.279	**2.43E-05**
**18**	0.044	-0.069	0.157	0.45	0.194	0.108	0.281	**2.16E-05**
**19**	0.036	-0.077	0.149	0.54	0.195	0.109	0.282	**1.97E-05**
**20**	0.027	-0.086	0.140	0.64	0.196	0.110	0.283	**1.85E-05**
**21**	0.017	-0.095	0.130	0.76	0.197	0.110	0.283	**1.81E-05**
**22**	0.007	-0.106	0.120	0.90	0.197	0.110	0.284	**1.85E-05**
**23**	-0.004	-0.117	0.108	0.94	0.197	0.110	0.284	**2.01E-05**
**24**	-0.016	-0.128	0.096	0.78	0.196	0.109	0.283	**2.32E-05**
**25**	-0.029	-0.141	0.083	0.62	0.194	0.107	0.282	**2.89E-05**
**26**	-0.042	-0.153	0.070	0.47	0.192	0.104	0.280	**3.89E-05**
**27**	-0.055	-0.167	0.056	0.33	0.189	0.100	0.278	**5.64E-05**
**28**	-0.069	-0.180	0.042	0.23	0.185	0.096	0.274	**8.77E-05**
**29**	-0.083	-0.193	0.028	0.15	0.180	0.090	0.270	**0.000145**
**30**	-0.096	-0.206	0.015	0.09	0.174	0.084	0.265	**0.00025**
**31**	-0.109	-0.219	0.002	0.06	0.168	0.077	0.259	**0.000445**
**32**	-0.121	-0.231	-0.011	**0.033642**	0.161	0.069	0.253	**0.000799**
**33**	-0.132	-0.242	-0.022	**0.020309**	0.154	0.061	0.246	**0.001423**
**34**	-0.142	-0.253	-0.032	**0.012475**	0.146	0.053	0.239	**0.002474**
**35**	-0.152	-0.262	-0.042	**0.007889**	0.139	0.046	0.232	**0.004154**
**36**	-0.160	-0.270	-0.050	**0.005178**	0.132	0.038	0.225	**0.006676**
**37**	-0.167	-0.277	-0.057	**0.003547**	0.125	0.031	0.218	**0.010216**
**38**	-0.173	-0.284	-0.063	**0.002542**	0.118	0.024	0.212	**0.014846**
**39**	-0.179	-0.289	-0.068	**0.001905**	0.113	0.019	0.207	**0.020478**
**40**	-0.183	-0.294	-0.073	**0.001493**	0.108	0.013	0.202	**0.026827**

Adjusted for maternal age, pre-pregnancy body mass index, race/ethnicity, marital status, educational status, health insurance ownership, parity, and mode of onset of labor. Statistically significant associations ($p<0.05$) are marked in bold.

Supplementary Table 5. Change in abdominal circumference *z*-score at 13-40 weeks gestation per one week increase in placental age acceleration in male and female fetuses.

	Male				Female			
		95% CI				95% CI		
Gestational week	Beta	Lower	Upper	*p*	Beta	Lower	Upper	*p*
**13**	0.051	-0.061	0.163	0.38	0.170	0.082	0.258	**0.000245**
**14**	0.046	-0.066	0.158	0.43	0.169	0.081	0.257	**0.000246**
**15**	0.040	-0.072	0.152	0.48	0.169	0.081	0.256	**0.000249**
**16**	0.034	-0.078	0.146	0.55	0.168	0.081	0.256	**0.000257**
**17**	0.028	-0.084	0.139	0.63	0.167	0.080	0.255	**0.000272**
**18**	0.021	-0.091	0.132	0.72	0.166	0.079	0.253	**0.000294**
**19**	0.013	-0.098	0.124	0.82	0.164	0.077	0.252	**0.000327**
**20**	0.005	-0.106	0.116	0.93	0.163	0.075	0.250	**0.000377**
**21**	-0.004	-0.114	0.107	0.95	0.160	0.073	0.247	**0.000451**
**22**	-0.012	-0.122	0.097	0.82	0.158	0.070	0.245	**0.000562**
**23**	-0.022	-0.131	0.088	0.70	0.154	0.067	0.242	**0.000729**
**24**	-0.031	-0.140	0.078	0.57	0.151	0.063	0.238	**0.000985**
**25**	-0.041	-0.150	0.067	0.46	0.146	0.059	0.234	**0.00138**
**26**	-0.051	-0.159	0.057	0.36	0.142	0.054	0.229	**0.001995**
**27**	-0.060	-0.168	0.047	0.27	0.136	0.048	0.225	**0.002951**
**28**	-0.070	-0.177	0.037	0.20	0.131	0.042	0.219	**0.004425**
**29**	-0.079	-0.185	0.028	0.15	0.125	0.036	0.214	**0.006654**
**30**	-0.087	-0.194	0.019	0.11	0.119	0.030	0.208	**0.009936**
**31**	-0.095	-0.201	0.011	0.08	0.113	0.024	0.203	**0.014598**
**32**	-0.102	-0.208	0.003	0.06	0.107	0.017	0.197	**0.020948**
**33**	-0.109	-0.214	-0.003	**0.04518**	0.102	0.011	0.192	**0.029203**
**34**	-0.114	-0.219	-0.009	**0.034911**	0.096	0.006	0.187	**0.039419**
**35**	-0.119	-0.224	-0.014	**0.027715**	0.091	0.000	0.182	0.05
**36**	-0.123	-0.228	-0.019	**0.022662**	0.087	-0.005	0.178	0.06
**37**	-0.127	-0.232	-0.022	**0.019113**	0.083	-0.009	0.174	0.08
**38**	-0.130	-0.234	-0.025	**0.016629**	0.079	-0.013	0.171	0.09
**39**	-0.132	-0.236	-0.027	**0.014917**	0.076	-0.016	0.168	0.11
**40**	-0.133	-0.238	-0.029	**0.013783**	0.073	-0.018	0.165	0.12

Adjusted for maternal age, pre-pregnancy body mass index, race/ethnicity, marital status, educational status, health insurance ownership, parity, and mode of onset of labor. Statistically significant associations ($p<0.05$) are marked in bold.

Supplementary Table 6. Change in humeral length *z*-score at 13-40 weeks gestation per one week increase in placental age acceleration in male and female fetuses.

	Male				Female			
		95% CI				95% CI		
**Gestational week**	**Beta**	**Lower**	**Upper**	***p***	**Beta**	**Lower**	**Upper**	***p***
**13**	0.054	-0.056	0.164	0.34	0.197	0.108	0.285	**2.75E-05**
**14**	0.053	-0.056	0.163	0.34	0.196	0.107	0.284	**2.97E-05**
**15**	0.052	-0.058	0.162	0.35	0.194	0.106	0.283	**3.27E-05**
**16**	0.051	-0.059	0.160	0.37	0.193	0.105	0.281	**3.69E-05**
**17**	0.049	-0.061	0.159	0.38	0.191	0.103	0.279	**4.26E-05**
**18**	0.047	-0.063	0.157	0.40	0.189	0.101	0.277	**5.1E-05**
**19**	0.045	-0.065	0.154	0.43	0.186	0.098	0.274	**6.36E-05**
**20**	0.042	-0.068	0.151	0.46	0.183	0.095	0.270	**8.34E-05**
**21**	0.038	-0.072	0.147	0.50	0.178	0.091	0.266	**0.000117**
**22**	0.033	-0.076	0.142	0.55	0.173	0.085	0.261	**0.000177**
**23**	0.028	-0.081	0.137	0.62	0.166	0.079	0.254	**0.000297**
**24**	0.021	-0.088	0.130	0.71	0.158	0.071	0.245	**0.000565**
**25**	0.012	-0.097	0.121	0.83	0.147	0.060	0.235	**0.001244**
**26**	0.002	-0.107	0.111	0.97	0.134	0.046	0.221	**0.003224**
**27**	-0.010	-0.119	0.099	0.86	0.116	0.029	0.203	**0.009786**
**28**	-0.024	-0.133	0.086	0.67	0.095	0.009	0.182	**0.033107**
**29**	-0.039	-0.149	0.071	0.49	0.070	-0.016	0.157	0.11
**30**	-0.055	-0.166	0.057	0.34	0.043	-0.043	0.129	0.33
**31**	-0.069	-0.181	0.043	0.23	0.015	-0.070	0.101	0.72
**32**	-0.081	-0.195	0.032	0.16	-0.010	-0.095	0.075	0.81
**33**	-0.091	-0.205	0.024	0.12	-0.032	-0.117	0.053	0.47
**34**	-0.098	-0.213	0.018	0.10	-0.048	-0.133	0.037	0.27
**35**	-0.102	-0.217	0.014	0.09	-0.058	-0.143	0.027	0.18
**36**	-0.103	-0.219	0.012	0.08	-0.062	-0.147	0.023	0.15
**37**	-0.103	-0.218	0.013	0.08	-0.060	-0.145	0.025	0.17
**38**	-0.100	-0.215	0.016	0.09	-0.052	-0.137	0.034	0.24
**39**	-0.094	-0.208	0.021	0.11	-0.036	-0.122	0.049	0.41
**40**	-0.084	-0.197	0.029	0.15	-0.014	-0.100	0.072	0.75

Adjusted for maternal age, pre-pregnancy body mass index, race/ethnicity, marital status, educational status, health insurance ownership, parity, and mode of onset of labor. Statistically significant associations ($p<0.05$) are marked in bold.

Supplementary Table 7. Change in femur length *z*-score at 13-40 weeks gestation per one week increase in placental age acceleration in male and female fetuses.

	Male				Female			
		95% CI				95% CI		
Gestational week	Beta	Lower	Upper	*p*	Beta	Lower	Upper	*p*
**13**	0.032	-0.076	0.141	0.56	0.179	0.087	0.271	**0.000219**
**14**	0.032	-0.076	0.141	0.56	0.179	0.087	0.271	**0.000218**
**15**	0.032	-0.077	0.141	0.56	0.178	0.086	0.270	**0.000219**
**16**	0.032	-0.077	0.140	0.57	0.177	0.086	0.269	**0.000222**
**17**	0.031	-0.078	0.140	0.58	0.177	0.086	0.268	**0.000227**
**18**	0.030	-0.078	0.139	0.58	0.176	0.085	0.266	**0.000237**
**19**	0.029	-0.079	0.138	0.60	0.174	0.084	0.265	**0.000252**
**20**	0.028	-0.081	0.137	0.61	0.173	0.082	0.263	**0.000274**
**21**	0.027	-0.082	0.135	0.63	0.170	0.081	0.260	**0.000308**
**22**	0.025	-0.084	0.133	0.66	0.168	0.078	0.257	**0.000362**
**23**	0.022	-0.087	0.131	0.69	0.164	0.075	0.253	**0.000451**
**24**	0.019	-0.090	0.127	0.74	0.159	0.071	0.248	**0.00061**
**25**	0.014	-0.094	0.122	0.80	0.153	0.065	0.241	**0.000927**
**26**	0.008	-0.099	0.116	0.88	0.144	0.056	0.232	**0.001654**
**27**	0.001	-0.107	0.109	0.99	0.132	0.045	0.219	**0.003672**
**28**	-0.009	-0.116	0.099	0.87	0.115	0.028	0.202	**0.010653**
**29**	-0.021	-0.128	0.086	0.70	0.092	0.005	0.179	**0.039932**
**30**	-0.035	-0.142	0.073	0.53	0.062	-0.025	0.149	0.16
**31**	-0.049	-0.157	0.059	0.37	0.027	-0.061	0.116	0.54
**32**	-0.062	-0.171	0.047	0.27	-0.008	-0.097	0.082	0.87
**33**	-0.072	-0.181	0.038	0.20	-0.038	-0.129	0.053	0.41
**34**	-0.078	-0.189	0.033	0.17	-0.060	-0.152	0.032	0.20
**35**	-0.082	-0.193	0.029	0.15	-0.073	-0.166	0.020	0.12
**36**	-0.083	-0.195	0.028	0.14	-0.077	-0.171	0.016	0.11
**37**	-0.084	-0.194	0.027	0.14	-0.073	-0.166	0.020	0.13
**38**	-0.082	-0.192	0.029	0.15	-0.059	-0.151	0.034	0.22
**39**	-0.076	-0.186	0.033	0.17	-0.033	-0.126	0.059	0.48
**40**	-0.067	-0.174	0.041	0.23	0.002	-0.089	0.093	0.96

Adjusted for maternal age, pre-pregnancy body mass index, race/ethnicity, marital status, educational status, health insurance ownership, parity, and mode of onset of labor. Statistically significant associations ($p<0.05$) are marked in bold.
